# State-of-the-Art Molecular Oncology in Brazil

**DOI:** 10.3390/ijms24119452

**Published:** 2023-05-29

**Authors:** Tiago Rodrigues

**Affiliations:** Centro de Ciências Naturais e Humanas (CCNH), Universidade Federal do ABC (UFABC), Santo André 09210-580, Brazil; tiago.rodrigues@ufabc.edu.br; Tel.: +55-(11)-4996-8371

Over the past few decades, the life expectancy of humankind has increased significantly due to advancements in life sciences and medical research, particularly given our increasing success in the epidemiological and pharmacological management of bacterial, fungi, and viral infections. However, the number of new cancer cases has increased due to this extended longevity. At present, cancer is one of the major causes of death worldwide; several factors contribute to the growing numbers in global cancer statistics, including genetic determinants, increased exposure to carcinogens, and the nutritional conditions of modern life.

Despite the noteworthy advances in the field, unresponsiveness, recidivism, side effects, and drug resistance are still obstacles to overcome in cancer treatment. Furthermore, the high cost of current therapies poses a significant problem for the public health systems in several countries, limiting patients’ access to the most recent drugs and therapeutic approaches. Conversely, significant advances have been made toward molecular aspects of tumor biology in the last decade(s), enabling researchers to foresee new avenues of cancer treatment with the aid of novel drugs/targets and innovative approaches, which must be brought to the attention of physicians and their patients.

Scientists worldwide are focused on understanding the molecular aspects of tumorigenesis, cancer progression, and metastasis. However, cancer treatment remains a very challenging task, as it depends on the intrinsic features of the disease (type, location, stage, mutations, responsiveness, and so on) and the conditions and variability among patients (comorbidities, immune system, and others). All these variables pose additional challenges for researchers and clinicians. Thus, the path from the bench to the bedside is long and includes multiple stages, from basic and preclinical studies to clinical trials.

It is well established that cancer cells exhibit a myriad of molecular alterations and adaptive transformations, enabling them to have unlimited proliferative capacity and invasiveness [1]. Furthermore, such alterations allow tumor cells to escape the regulatory mechanisms of cell growth/death and evade recognition/destruction by the immune system. All these features are elicited or accompanied by metabolic reprogramming, gene/protein expression changes, and alterations in several signaling pathways. Based on this, a vast therapeutic arsenal is now available for cancer treatment, varying from old cytotoxic drugs to the current personal precision oncology [2]. Cancer therapy now includes conventional chemotherapy, photodynamic therapy, inhibitors of angiogenesis, targeted therapy, immunotherapy, CAR-T cell therapy, oncolytic viruses, and antitumor vaccines [3,4]. Additionally, combined therapies, the development of second- and third-generation drugs, nanotechnological approaches, and drug repurposing have arisen as possible strategies to improve drug responsiveness and overcome drug resistance [5,6,7,8,9]. At last, the possibility of genome edition/correction by the CRISPR-Cas9 methodology represents a promising tool to be employed in treating cancer in the near future [10].

Notably, the improvement of analytical methods and experimental capabilities catalyzed the current advancements in the field of molecular oncology. These advancements are pivotal to discovering novel targets and drugs for cancer treatment. Among such novel strategies, the screening of new molecules represents a prospective approach for drug discovery, helping to overcome drug resistance [11,12,13]. Furthermore, identifying oncogenes and their regulation by miRNAs and epigenetic alterations enable the discovery of new biomarkers for cancer diagnosis, paving the way for new therapeutic opportunities [14,15]. The modulation of PD-L1 expression/activity is also a promising therapeutic strategy [16].

I am, therefore, pleased to introduce the papers published in this Special Issue, “State-of-the-Art Molecular Oncology in Brazil”. These manuscripts represent a sampling of the ongoing studies conducted in Brazil in the field of cancer research, and are also a tribute to the high-quality research developed in the country in spite of the severe restrictions imposed on research funding in recent years. I would also like to thank all authors for their valuable contributions, and the reviewers who took part in the peer-review process. Finally, I hope the audience worldwide will enjoy the new information and discussions provided by this Special Issue.
